# OmpR Indirectly Regulates Biosynthesis of Xenocoumacin 1 in *Xenorhabdus nematophila*

**DOI:** 10.3390/microorganisms13061360

**Published:** 2025-06-11

**Authors:** Yunfei Han, Xintong Zhao, Mengru He, Shujing Zhang, Gaijuan Tang, Yonghong Wang

**Affiliations:** 1 Key Laboratory of Plant Protection Resources and Pest Management, Ministry of Education, Shaanxi Biopesticide Engineering & Technology Research Center, College of Plant Protection, Northwest A&F University, Yangling 712100, China; hanyunfei@nwafu.edu.cn (Y.H.);; 2 Key Laboratory of Green Prevention and Control of Tropical Plant Diseases and Pests, Ministry of Education, School of Tropical Agriculture and Forestry (School of Agricultural and Rural Affairs, School of Rural Revitalization), Hainan University, Haikou 570228, China; 3 Hybrid Rapeseed Research Center of Shaanxi Province, Yangling 712100, China

**Keywords:** *Xenorhabdus nematophila*, transcriptome, xenocoumacin 1, OmpR, EMSA

## Abstract

*Xenorhabdus nematophila* has excellent potential for application in both medicine and agriculture due to its various active secondary metabolites. The transcriptional regulator OmpR negatively regulates Xenocoumacin 1 (Xcn1), which has wide antimicrobial activity. Here, we expressed and purified OmpR and verified its binding activities to promoters via an electrophoretic mobility shift assay. RNA sequencing was used to analyze the relevance and difference of differentially expressed genes between *X. nematophila* and its mutant *ΔompR*. Compared with the WT, 1127 differentially expressed genes were found in *ΔompR*, while 4150 co-expressed genes were detected. RT-qPCR data validated the RNA-seq results with 20 randomly selected genes. OmpR positively regulates the process of porphyrin metabolism, quorum sensing, β-Lactam resistance and glyoxylate and dicarboxylate metabolism, while negatively regulating the phosphotransferase system, two-component system and bacterial chemotaxis. OmpR indirectly regulates the biosynthesis of Xcn1 by positively regulating the process of glyoxylate metabolism, which consumes energy and precursors, and negatively regulates biomacromolecules biosynthesis, which provides energy and precursors. Overall, this work revealed the indirect effects of OmpR on the biosynthesis of Xcn1, serving as a foundation for future research into the intricate regulatory network of *X. nematophila*.

## 1. Introduction

*Xenorhabdus nematophila*, a Gram-negative bacterium classified as *Morganellaceae*, forms a mutualistic symbiosis with the entomopathogenic nematode *Steinernema* [1,2]. Both the bacterium *Xenorhabdus* and the nematode *Steinernema* generate a variety of specialized metabolites during the life cycle of the mutualistic symbiosis that act synergistically to ensure their proliferation and reproduction [3,4]. *Xenorhabdus* has excellent potential for application in both medicine and agriculture. Particularly, compounds produced by *Xenorhabdus* have a broad spectrum of biological activity, inhibiting the growth of bacteria [5], fungi [6], oomycetes [7], acarine [8], nematode [9], insects [10] and protozoa [11].

Xenocoumacin 1 (Xcn1) is a significant antimicrobial compound produced by *X. nematophila* and has wide prospects for application in sustainable agricultural development. In the biosynthetic gene cluster of Xcn1, *xcnA-L* genes contribute to Xcn1 biosynthesis and *xcnMN* genes act on Xcn1 to transform its analogs [4,12]. Acetyl coenzyme A and 1, 3-bisphosphoglycerate are important precursors of Xcn1 biosynthesis [4]. Seven transcriptional regulatory factors regulate Xcn1 biosynthesis: FliZ [13], Hfq [2] and Lrp [3] regulate Xcn1 positively, while CpxR [14], LeuO [3], LrhA [15] and OmpR [16] regulate it negatively.

The production of diverse active secondary metabolites is attributed to the environment in which *X. nematophila* exists. Two-component systems, including CpxA/CpxR and EnvZ/OmpR, play a crucial role in responding to nematodes and the environment. The sensor histidine kinases CpxA and EnvZ sense changes in pH and osmolarity, and phosphorylate the regulators CpxR and OmpR to govern target genes [14,17,18]. OmpR, a vital transcriptional regulator that governs over a hundred genes, is responsible for controlling antibiotic production and the formation of flagella and exoenzymes in *X. nematophila* [19,20]. OmpR binds to the promoter region to regulate target genes directly. There is a binding site of OmpR in the promoter region of the *opnP* gene in *X. nematophila* [21]. The ability of phosphorylated OmpR to bind to promoters was significantly higher than that of unphosphorylated OmpR [22]. Crosstalk between CpxA and OmpR or between EnvZ and CpxR, might happen when there is a lack of cognate signaling partners [23]. Interestingly, OmpR and CpxR occasionally bind to the same promoter simultaneously [24]. Although CpxR and OmpR jointly regulate some physiological processes, whether there is a direct regulation between these two regulators is still unknown. OmpR negatively governs the production of Xcn1 by negatively regulating *xcnA-L* and positively regulating *xcnMN* expression [16]. However, it is unclear which regulatory factor directly regulates the biosynthesis of Xcn1 by binding to the promoter region of the *xcnA* gene.

In this work, the transcriptional regulation factor OmpR was purified, and the DNA-binding ability in promoters was determined. Furthermore, *ompR* gene deletion mutant strains were constructed from *X. nematophila* YL001, and differentially expressed genes were analyzed via enrichment analysis. In addition, a model for regulating Xcn1 biosynthesis via OmpR was preliminarily established.

## 2. Materials and Methods

### 2.1. Strains and Their Growth Conditions

*Xenorhabdus nematophila* YL001 was isolated from its symbiotic nematode, *Steinernema* sp. YL001, which was obtained from the soil of Yangling, China [25,26]. The morphological and molecular characteristics of *X. nematophila* YL001 have been identified [26]. Detailed information on the strains and plasmids utilized in this study is displayed in Table 1.

*X. nematophila* and *E. coli* were grown in Luria–Bertani (LB) medium at 28 °C and 37 °C, respectively. Agar and antibiotics were supplied to the LB medium if desired. Kanamycin is used to culture *E. coli* containing vectors for heterologous expression of OmpR. Chloramphenicol is used to screen for *E. coli* containing suicide vectors for *ompR* knockout. Sucrose and kanamycin were used to screen for *ΔompR*.

### 2.2. DNA Manipulation

Genomic DNA and plasmid were extracted individually using the Rapid Bacterial Genomic DNA Isolation Kit (Sangon, Shanghai, China) and the HiPure Plasmid EF Mini Kit (Magen, Foshan, China) according to the manufacturer’s instruction manual. Polymerase chain reaction (PCR) amplification was conducted with the help of Hieff Canace^®^ Plus High-Fidelity DNA Polymerase (Yeasen, Shanghai, China) and primers listed in Tables S1–S3. The DNA fragments produced by PCR were purified utilizing the SanPrep Column DNA Gel Extraction Kit (Sangon, China). Recombinant plasmids were structured by the Hieff Clone^®^ Universal One Step Cloning Kit (Yeasen, China). Primers for PCR amplification were designed by Primer Premier 5.0 software [27]. Recombinant plasmids and mutant strains were verified by DNA sequencing (AuGCT, Beijing, China).

### 2.3. Protein Expression and Purification

Protein expression and purification were conducted based on published methods [28,29]. In detail, fragments containing homologous arms for recombinant vector construction were amplified from the chromosomal DNA of *X. nematophila* YL001. Primers for heterologous expression of regulators are shown in Table S1. Fragments of OmpR-N_6His_ were cloned into pET28a plasmids that were cleaved using BamHI (Takara, Osaka, Japan) and NdeI (Takara, Osaka, Japan), creating pET28a-OmpR. Recombinant vectors were transformed into *E. coli* DH5α for reproduction. After DNA sequencing, recombinant plasmids were transformed into *E. coli* BL21 (DE3). *E. coli* BL21 (DE3) was cultured at 37 °C and 200 rpm until an optical density up to 1 at 600 nm. After isopropyl-beta-D-thiogalactopyranoside (IPTG) was supplemented with concentrations of 0.5 mM, cultures were shaken overnight at 16 °C and 180 rpm.

Cells were collected by centrifugation and resuspended in lysis buffer (50 mM NaH_2_PO_4_, 300 mM NaCl, pH 8.0). Subsequently, lysozyme (1 mg/mL final), AEBSF (2 mM final), bestatin (0.13 mM final) and leupeptin (10 μM final) were added to the cell suspension and incubated on ice for 30 min. Cells were lysed by passage through the Ultrasonic cell pulverizer (Scientz Biotechnology, Ningbo, China) six times, and the lysate was centrifuged for 30 min at 4 °C and 10,000 rpm to collect the supernatant. The supernatant was subjected to BeyoGold™ His-tag Purification Resin (Beyotime, Shanghai, China) for protein binding. Purified protein was obtained by washing and elution with imidazole solutions in gradient concentrations (10~100 mM imidazole, 50 mM NaH_2_PO_4_, 300 mM NaCl, pH 8.0). Eluted fractions were sampled and analyzed by sodium dodecyl sulfate-polyacrylamide gel electrophoresis (SDS-PAGE). Purified OmpR-N_6His_ was represented by SDS-PAGE and captured by Xiaomi 11 LE. Purified protein was concentrated using the Microsep Advance Centrifugal Device (Pall Corporation, Port Washington, NY, USA) at 8000 rpm and 4 °C and stored at −20 °C.

### 2.4. Electrophoretic Mobility Shift Assay (EMSA)

EMSAs were carried out according to the reported method [30,31]. In detail, DNA probes were amplified from the chromosomal DNA of *X. nematophila* YL001 by PCR using the primers listed in Table S2. Concentrations of purified protein and DNA probe were determined by the K5800 ultramicro spectrophotometer (KAIAO, Beijing, China). DNA probes, purified protein and 5× reaction buffer (250 mM Tris, 250 mM KCl, 0.5 mM DTT, 50 mM MgCl_2_, 25% glycerol, pH 8.0) were incubated for 30 min at room temperature. The reaction mixture was supplemented with 5× EMSA loading buffer (Beyotime, Shanghai, China) to stop the reaction and subsequently analyzed by 6% native acrylamide gel electrophoresis that was performed at 90 V in 0.5× TBE buffer (45 mM Tris-HCl, 45 mM boric acid, pH 8.0) at 4 °C. Gels were dyed with Ultra GelRed (Vazyme, Nanjing, China), and the results were captured by GenoSens transilluminator (Clinx, Shanghai, China).

### 2.5. Construction of Mutant Strains

For recombinant suicide vector construction, fragments that contained homologous arms upstream and downstream of *ompR* were amplified individually from the chromosomal DNA of *X. nematophila* YL001 by PCR using the primers in Table S3. Kanamycin-resistant fragments containing homologous arms were separately amplified from pET28a. Primers for the construction and verification of mutant strains are shown in Table S3. The upstream, downstream and kanamycin-resistant fragment were connected and cloned into pDM4 plasmids that were cleaved using restriction endonuclease SphI (Takara, Japan) and SacI (Takara, Japan). Recombinant plasmids were transformed into *E. coli* S17-1λ*pir* and conjugally transferred into the *X. nematophila* YL001 [14,16]. Successful recombinants were identified in the LB medium containing sucrose, ampicillin and kanamycin.

### 2.6. RNA Isolation and Library Preparation

RNA was extracted with Trizol after the bacteria used for RNA extraction were grown in the LB medium to the logarithmic growth phase at 28 °C and 180 rpm. Genomic DNA was disintegrated with the help of DNase Ⅰ without RNase. The integrity and quality of RNA were evaluated utilizing the RNA Nano 6000 Assay Kit of the Bioanalyzer 2100 system (Agilent Technologies, Santa Clara, CA, USA). The purification of mRNA from total RNA was performed by using probes to remove rRNA. Subsequently, random hexamer primers and M-MuLV Reverse Transcriptase were employed to synthesize the first strand cDNA, and subsequently, RNaseH was used to degrade the RNA. The second strand of cDNA was synthesized using dUTP to replace the dTTP of dNTP as the raw material in the DNA polymerase I system. Blunt ends were produced by exonuclease/polymerase activities from the remaining overhangs. Adaptors with hairpin loop structures were ligated to prepare for hybridization after they had been adenylated on the 3′ ends of DNA fragments. Then, USER Enzyme was used to degrade the second strand of cDNA containing U. Purification of the library fragments with the AMPure XP system (Beckman Coulter, Brea, CA, USA) was performed to select the preferred cDNA fragments, which ranged from 370 to 420 bp in length. Products were purified by the AMPure XP system after PCR performed with Phusion High-Fidelity DNA polymerase, Universal PCR primers and Index (X) primers. Library quality was assessed on the Agilent Bioanalyzer 2100 system. As per the manufacturer’s instructions, the index-coded samples were clustered on a cBot Cluster Generation System using the TruSeq PE Cluster Kit v3-cBot-HS (Illumia, San Diego, CA, USA). After cluster generation, the purified cDNA library was sequenced using the Illumina Novaseq platform, and then 150 bp paired-end reads were generated.

### 2.7. Bioinformatics Analysis

First, in-house perl scripts were used to process raw data (raw reads) in the fastq format. Clean data (clean reads) were obtained by removing reads containing adapters, reads containing N bases and low-quality reads from raw data. At the same time, Q20, Q30 and GC content of the clean data were calculated. All the downstream analyses were based on clean data of high quality. Next, Bowtie 2 (2.2.3) was used to map the clean reads to the *X. nematophila* YL001 transcriptome [32]. The number of reads mapped to each gene was counted by HTSeq (version 0.6.1) [33]. And then the FPKM (Fragments Per Kilobase of transcript per Million mapped reads) of each gene was calculated based on the length of the gene and read counts mapped to this gene. Differential expression analysis of groups was performed using the DESeq R package (1.18.0) [34]. Gene Ontology (GO) enrichment analysis of DEGs was implemented by the GOseq R package, in which gene length bias was corrected [35]. The statistical enrichment of genes that are differentially expressed in KEGG pathways was tested using KOBAS software (3.0) [36,37].

### 2.8. Reverse Transcription Quantitative PCR (RT-qPCR)

Total RNA was extracted using the FastPure Cell/Tissue Total RNA Isolation Kit (Vazyme, Nanjing, China). HiScript III RT SuperMix for qPCR (Vazyme, China) was used to produce cDNA, and the FastHS SYBR QPCR mixture (ALLMEEK, Beijing, China) was used to amplify the tested genes. RT-qPCR amplifications were performed using the CFX96 Touch gene amplification instrument (BIO-RAD, Hercules, CA, USA). The relative expression levels were calculated using the 2^−ΔΔCt^ method [38] and the housekeeping gene *recA* [14] as the internal control to normalize the transcript levels of the tested genes.

## 3. Results and Discussion

### 3.1. Validation of Binding Activity of OmpR in Promoters

**Figure 1:**
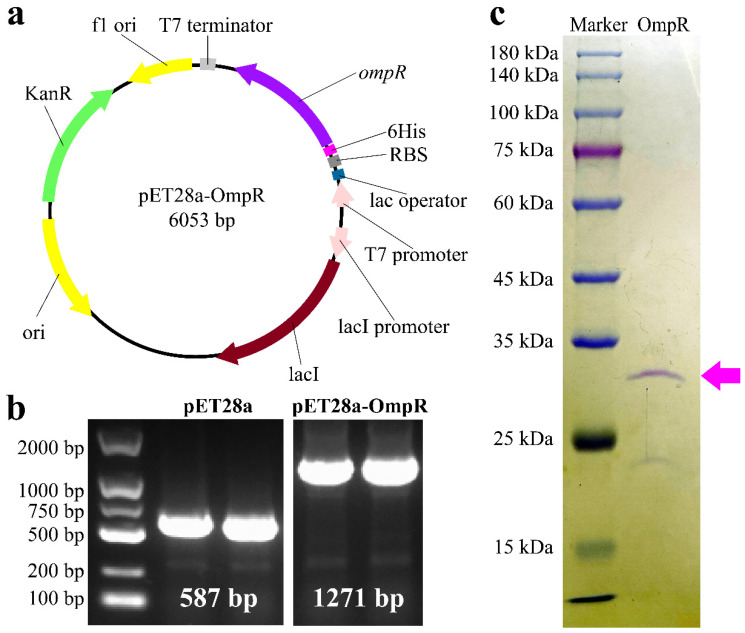
Heterologous expression of OmpR of *X. nematophila* YL001. (**a**) Vector containing *ompR* for OmpR labeled with 6 histidine. (**b**) Verification of recombinant vectors for protein expression by PCR using primers 28a-F/R. (**c**) Purified protein OmpR-N_6His_ (29.52 kDa) pointed by purple arrows.

**Figure 2:**
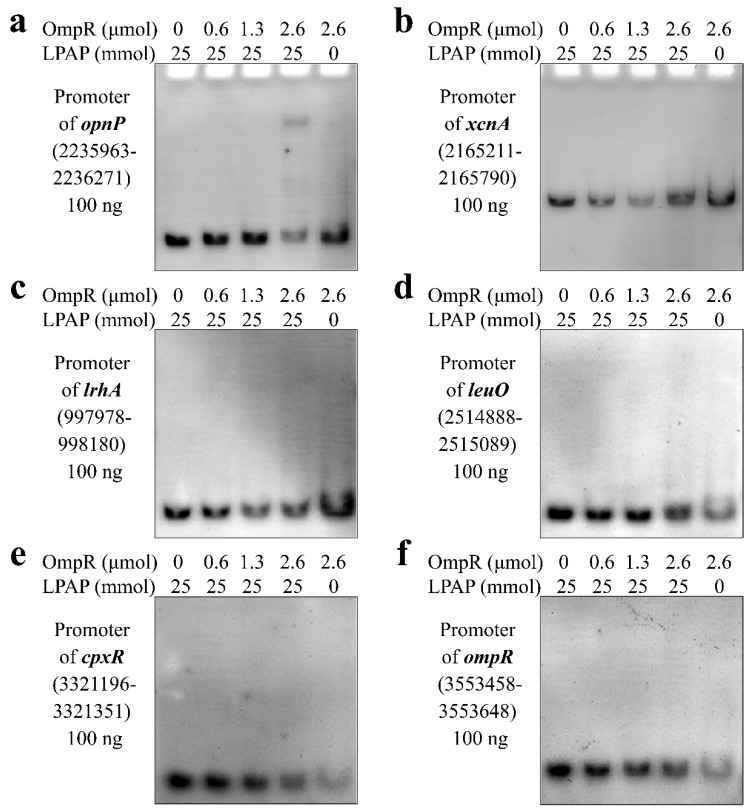
Verification of OmpR binding to promoter of various genes. LPAP, lithium potassium acetyl phosphate. The numbers in brackets represent the position of DNA in the genome of *X. nematophila* YL001. (**a**) OmpR bound to the promoter of *opnP*. (**b**) OmpR did not bind to the promoter of *xcnA*. (**c**) OmpR did not bind to the promoter of *lrhA*. (**d**) OmpR did not bind to the promoter of *leuO*. (**e**) OmpR did not bind to the promoter of *cpxR*. (**f**) OmpR did not bind to the promoter of *ompR*.

Recombinant vector pET28a-OmpR was constructed and verified to express OmpR (Figure 1a,b). OmpR was purified by imidazole solution and displayed by polyacrylamide gel electrophoresis (Figure 1c). The binding activity of active OmpR was determined by an EMSA. The promoter of *opnP* (Figure 2a) was the positive control according to published data [21]. A previous study suggested that OmpR negatively regulated the production of Xcn1 [16].

In this work, we found that OmpR was indirectly regulated because it could not bind to the promoter region of *xcnA* (Figure 2b). It indicated that OmpR could not directly regulate the expression of the *xcnA* gene. OmpR did not bind the promoter of *lrhA* in this study (Figure 2c) but bound to the promoter in *Pantoea alhagi* [39], which indicates the different regulatory mechanisms of OmpR between *X. nematophila* and *P. alhagi*. LeuO, CpxR and OmpR were negative regulatory factors for Xcn1 production [3,14,16]. Regretfully, OmpR did not directly regulate *leuO* (Figure 2d) and *cpxR* (Figure 2e) because no binding to the promoter region probe was detected, which demonstrated that OmpR cannot directly regulate two regulatory factors, LeuO and CpxR. Moreover, OmpR did not directly regulate its gene expression, as no obstruction was detected (Figure 2f).

### 3.2. Construction and Verification of Strains ΔompR

**Figure 3:**
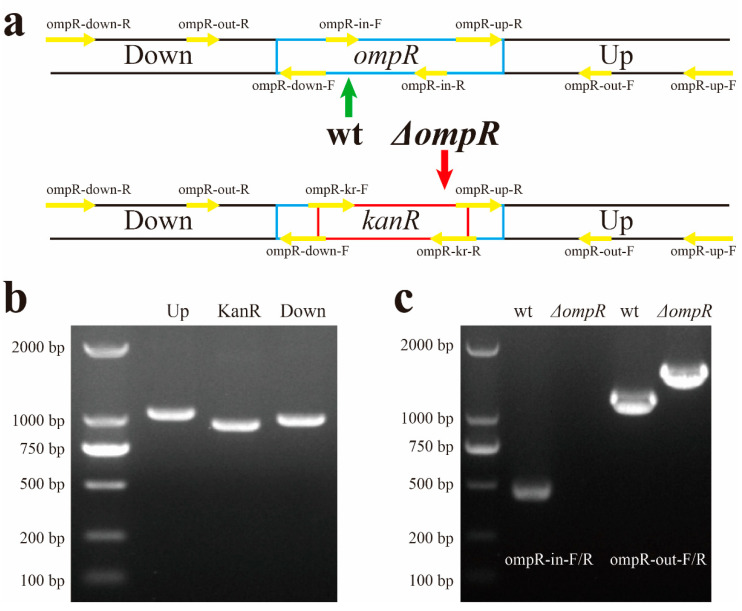
Construction and verification of strains *ΔompR*. (**a**) Graphical overview of gene knockout, including primers (yellow arrow) and location of *ompR* (cyan box) and *kanR* (red box). (**b**) Fragments for construction of *ΔompR*. Up: upstream of *ompR* (1038 bp); KanR: kanamycin-resistant cassette (941 bp); Down: downstream of *ompR* (990 bp). (**c**) Identification of the *ΔompR* with internal and external primers: 454 bp PCR products were generated by ompR-in-F/R with wild type (wt) as template, while no products were generated with *ΔompR* as template; 1118 bp PCR products were generated by primers ompR-out-F/R with wt as template; 1424 bp PCR products were generated with *ΔompR* as template.

To investigate the regulation mechanism of OmpR in *X. nematophila* on biosynthesis of Xcn1, *ΔompR* was constructed by replacing the *ompR* gene with the *kanR* box separately (Figure 3a). Thus, 1038 bp fragments upstream of *ompR*, 941 bp kanamycin-resistant cassette fragments and 990 bp fragments downstream of *ompR* were multiplied for the construction of the gene knockout vector (Figure 3b). Internal and external primers were used to verify *ΔompR* (Figure 3c). In *ΔompR*, no products were generated by primers ompR-in-F/R, and a longer fragment was generated by primers ompR-out-F/R compared to the wild type (wt).

### 3.3. Quantitative Analysis and Differential Expression Analysis of Genes

**Figure 4:**
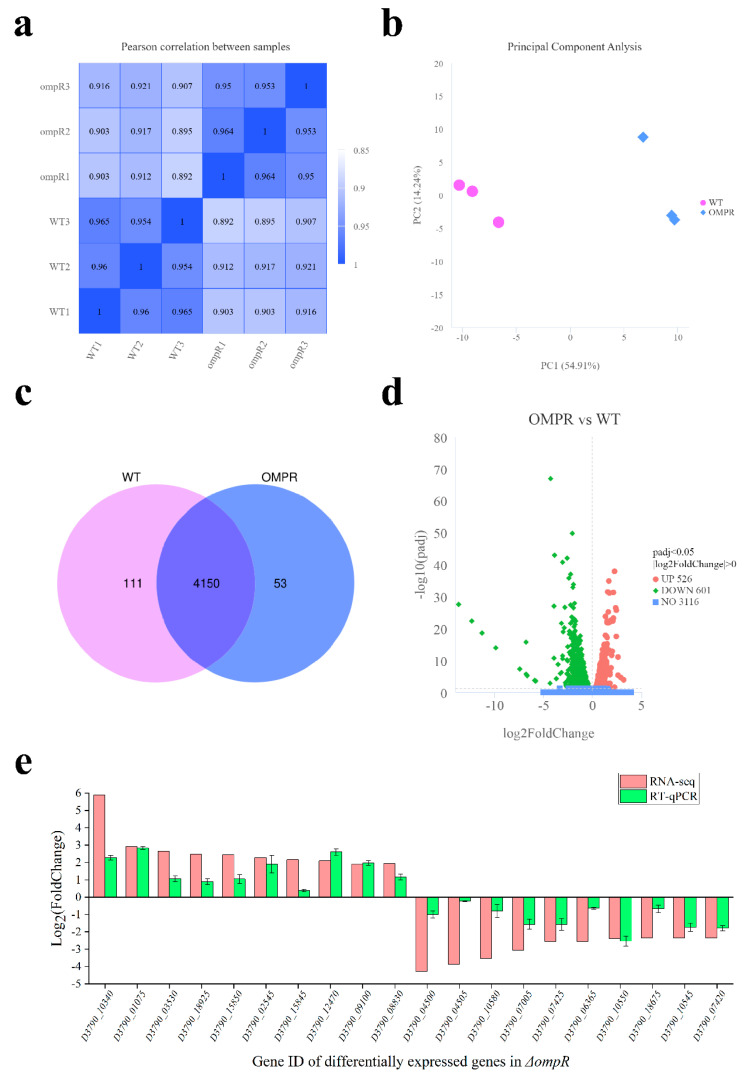
Correlation and differentially expressed genes analysis based on RNA-Seq. (**a**) Correlation of gene expression levels between *X. nematophila* YL001 (WT) and *ΔompR*. (**b**) Principal component analysis (PCA) of gene expression levels between WT and *ΔompR*. (**c**) Venn diagram of co-expressed genes between WT and *ΔompR*. (**d**) Volcano map of differentially expressed genes between *ΔompR* and WT. (**e**) Differentially expressed genes of RNA-Seq in *ΔompR* were validated by RT-qPCR.

The Pearson correlation R^2^ between samples of strains is displayed, illustrating that replicates of samples within groups were reliable (Figure 4a). The two-dimensional plot reveals that different individuals from the same group cluster together through principal component analysis (PCA), demonstrating that the sum of technical and biological variation is significantly smaller than the changes between *ΔompR* and WT (Figure 4b). In this experiment, 4150 co-expressed genes were detected (Figure 4c). Compared with the WT (|log2 Fold Change| > 0, p adj < 0.05), a total of 526 up-regulated genes and 601 down-regulated genes were detected in *ΔompR* (Figure 4d). Randomly, twenty significantly differentially expressed genes (DEGs) were selected to perform an RT-qPCR experiment for validating the data of RNA-Seq (Figure 4e). The RT-qPCR results of the DEGs were in accordance with the results observed in the RNA-seq data.

### 3.4. Enrichment Analysis of Differentially Expressed Genes in ΔompR

**Figure 5:**
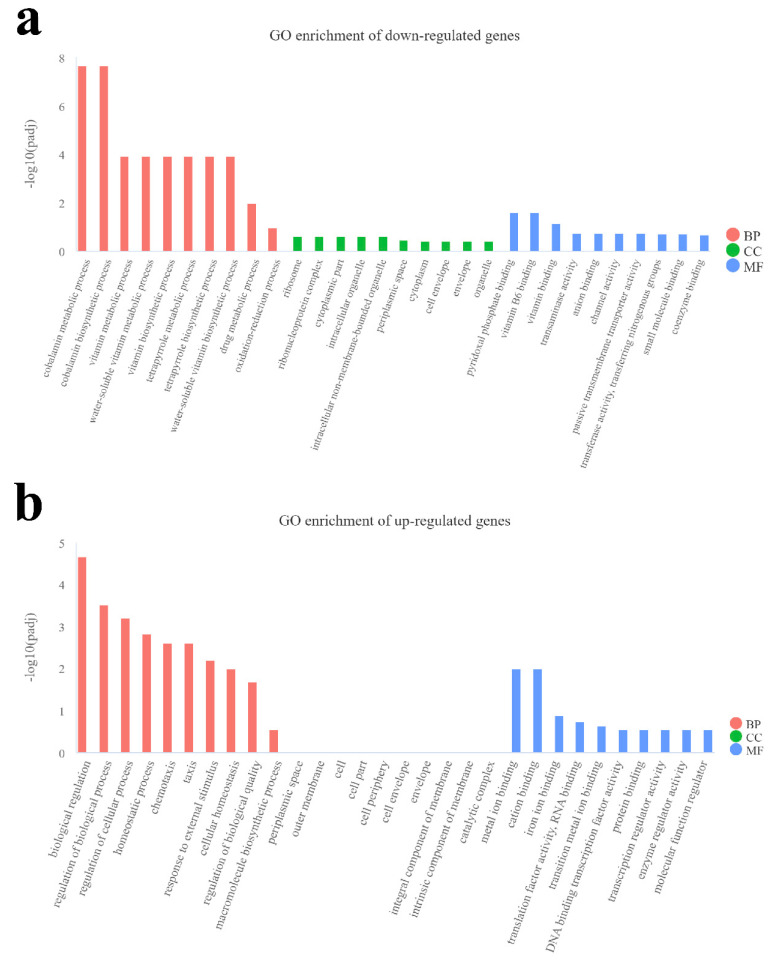
Gene Ontology (GO) enrichment analysis of differentially expressed genes. Abscissa represents the GO term that was significantly enriched, and ordinate represents the significance level of the GO term enriched. A higher value of ordinate indicates that the term was enriched more significantly. Different colors represent the three subclasses: biological process (BP, red), cellular component (CC, green) and molecular function (MF, blue). (**a**) Enriched terms of decreased genes in the *ΔompR*. (**b**) Enriched terms of increased genes in the *ΔompR*.

From the GO enrichment analysis results, the top 30 enriched terms were selected to draw a bar chart for display. Among the decreased expression genes in *ΔompR*, nine terms of biological process, including metabolic and biosynthetic processes of cobalamin, vitamin and tetrapyrrole, and two terms of molecular function, including pyridoxal phosphate binding and vitamin B6 binding, were significantly (*p* < 0.05) enriched (Figure 5a). Among the increased expression genes in *ΔompR*, 33 terms of biological process, including the regulation of biological process, chemotaxis, response to external stimulus and the regulation of macromolecule metabolic process, among others, and 2 terms of molecular function, including metal ion binding, were significantly (*p* < 0.05) enriched (Figure 5b).

**Figure 6:**
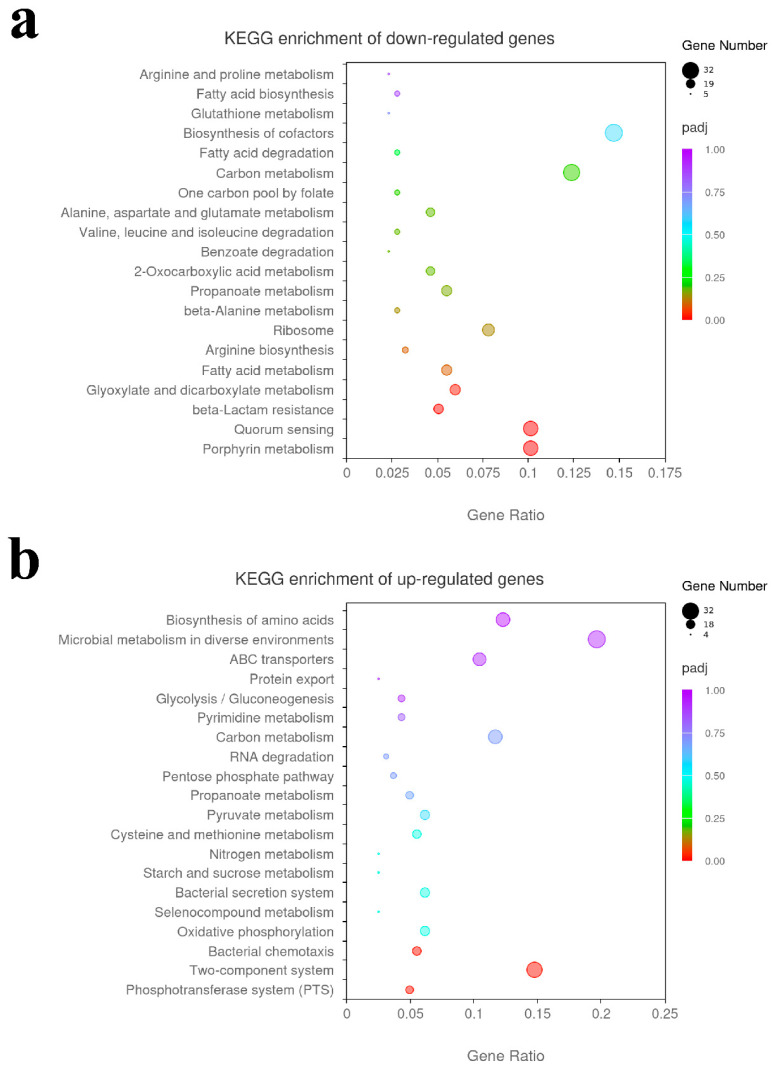
KEGG pathway enrichment analysis of differentially expressed genes. The abscissa is the ratio of the number of DEGs annotated to the KEGG pathway and the total number of DEGs, and the ordinate is the KEGG pathway. The number of DEGs was displayed by the size of the dots. The color of the dots gradually changes from purple to red, indicating that the pathway was enriched more significantly. (**a**) KEGG pathway enrichment analysis of down-regulated genes in *ΔompR*. (**b**) KEGG pathway enrichment analysis of up-regulated genes in *ΔompR*.

The top 20 enriched pathways were selected to draw a dot diagram to display the Kyoto Encyclopedia of Genes and Genomes (KEGG) pathway enrichment analysis of the DEGs. Decreased expression genes in *ΔompR* were enriched in four pathways: porphyrin metabolism, including 22 genes; quorum sensing, including 22 genes; beta-Lactam resistance, including 11 genes; and glyoxylate and dicarboxylate metabolism, including 13 genes (Figure 6a). Increased expression genes in *ΔompR* were enriched in three pathways: the phosphotransferase system, including 8 genes; the two-component system, including 24 genes; and bacterial chemotaxis, including 9 genes (Figure 6b).

**Figure 7:**
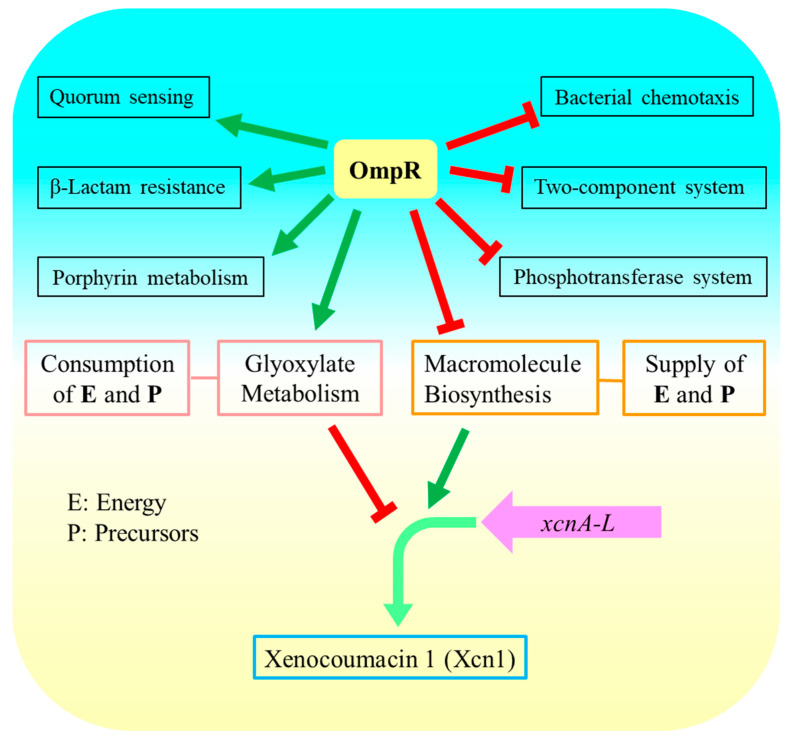
OmpR regulates biosynthesis of Xcn1 indirectly in *X. nematophila.* Green arrows represent positive regulation, and red T-shaped arrows represent negative regulation.

The biosynthesis of Xcn1 required energy and precursor 1,3-bisphosphoglycerate [4]. OmpR negatively regulated Xcn1 in *X. nematophila* [16]. GO enrichment found that the macromolecule biosynthetic process, including 41 genes, was negatively regulated by OmpR. It is presumed that OmpR regulates biosynthesis of Xcn1 by negatively regulating biomacromolecules biosynthesis (Figure 5b). KEGG pathway enrichment analysis found that down-regulated genes were significantly enriched in the glyoxylate and dicarboxylate metabolism pathway in *ΔompR*. Glyoxylate and dicarboxylate metabolism pathway consumed the energy and Xcn1 biosynthesis precursors. Therefore, we supposed that OmpR regulates the biosynthesis of Xcn1 by positively regulating the process of glyoxylate metabolism (Figure 7).

## 4. Conclusions

OmpR positively regulates the process of porphyrin metabolism, quorum sensing, β-Lactam resistance and glyoxylate and dicarboxylate metabolism, while negatively regulating the phosphotransferase system, the two-component system and bacterial chemotaxis. OmpR indirectly regulates the biosynthesis of Xcn1 by positively regulating the process of glyoxylate metabolism, which consumes energy and precursors, and negatively regulates biomacromolecules biosynthesis, which provides energy and precursors (Figure 7).

## Figures and Tables

**Table 1:** Bacterial strains and plasmids used in this study.

Strains	Relevant Characteristics	Source
***Xenorhabdus nematophila***		
YL001	Wild type; Amp^r^	Laboratory stock
*ΔompR*	*ompR* of *X. nematophila* YL001 was replaced by Km^r^	This study
***Escherichia coli***		
S17-1λ*pir*	Donor strain for conjugations	AngYuBio
DH5α	Strain for the reproduction of recombinant pET28a	AngYuBio
BL21(DE3)	Strain for protein expression	AngYuBio
**Plasmids**		
pDM4	Suicide vector; Cm^r^, *sacB*	Laboratory stock
pDM4-*ompR*-Km^r^	Recombinant plasmid pDM4 for *ΔompR* construction	This study
pET28a	Source of Km^r^ gene; Vector for protein expression	Laboratory stock
pET28a-OmpR	Protein expression vector of OmpR-N_6His_	This study

Note: Amp^r^, Ampicillin resistance; Km^r^, Kanamycin resistance; Cm^r^, Chloramphenicol resistance.

## Data Availability

The original contributions presented in this study are included in the article/Supplementary Material. Further inquiries can be directed to the corresponding authors.

## Supplementary Materials

The following supporting information can be downloaded at https://www.mdpi.com/article/10.3390/microorganisms13061360/s1, Table S1. Primers for protein expression; Table S2. Primers for electrophoretic mobility shift assay; Table S3. Primers for construction and verification of mutant strains. Supplementary Excels: Enrichment analysis results of differentially expressed genes in *ΔompR*.
