# Supplementary material for: OmpR Indirectly Regulates Biosynthesis of Xenocoumacin 1 in Xenorhabdus nematophila
Source: Microorganisms. 2025 Jun 11;13(6):1360. doi: 10.3390/microorganisms13061360 (PMC12196045; doi:10.3390/microorganisms13061360)
Supplement: Supplementary file 1 [file microorganisms-13-01360-s001.zip › Supplementary materials.pdf]

Supplementary Tables

Table S1 Primers for protein expression

Primers	Sequence (5'-3')	Notes
ompR-n-F	GGCCTGGTGCCGCGCGGCAGCCATATGC AAGAGAATTATAAGATCCTTGT	Expression of OmpR-N_{6His}
ompR-n-R	TGTCGACGGAGCTCGAATTCGGATCCTC ATACTTTATTTCCATCAGGGATAAAAAC	
28a-F	GGGGTTATGCTAGTTATTGC	Validation of recombinant pET28a plasmid
28a-R	CCCAGTAGTAGGTTGAGGC	

Table S2 Primers for electrophoretic mobility shift assay

Primers	Sequence (5'-3')	Notes
PcpxR156-F	GCATTAAATTTCTCCGCTTTC	Promoter region of *cpxR*
PcpxR156-R	TAATTCTTCCTCCAAGAGCAA	
PompR191-F	CCGGCCTTTGGTAATAAATCAGG	Promoter region of *ompR*
PompR191-R	GTTTCAGCTCCCAAGATGCT	
PxcnA580-F	CTCGTTTTGGTGAAGAGCAG	Promoter region of *xcnA*
PxcnA580-R	TACGTCTCGTAACGCTTCAT	
PopnP309-F	TGAATCAGCTCAATAAATAAGCTAAATT	Promoter region of *opnP*
PopnP309-R	TTGTTATTACCTCATTGGTGTTATTTAG	
PleuO202-F	TACGGCTACGTAAATAATAAAGCAT	Promoter region of *leuO*
PleuO202-R	TATTTCACTCCACTAAACTTATCGCA	
PlrhA203-F	TTTGGATTGCATAAATTTTGTTAAGA	Promoter region of *lrhA*
PlrhA203-R	CATTGTTTATTCATCACTTTTTTTTG	

Table S3 Primers for construction and verification of mutant strains

Primers	Sequence (5’-3’)	Notes
ompR-up-F	AGTGGGGCCCTTCTAGATAGATCTTGCATGCGATGCGACTTAAAGATTCATTCA	5’ upstream region of *ompR*
ompR-up-R	GTTTTTCTAAGAATCACCTCAGGCGCATATCATCA	
ompR-down-F	ACTGAGCGTCAGTATATTCAGACCGTCTGGGGAT	3’ downstream region of *ompR*
ompR-down-R	GATAACAATTTGTGGAATCCCGGGAGAGCTCAACATGACGGAACCGCT	
ompR-kr-F	CGGTCTGAATATACTGACGCTCAGTGGAACGA	Kanamycin-resistant cassette
ompR-kr-R	TATGCGCCTGAGGTGATTCTTAGAAAAACTCATCGAGCATC	
ompR-in-F	ATTTATCCCGTGACAGCG	Internal fragment of *ompR*
ompR-in-R	GCGGCAAATGCAGAACAG	
ompR-out-F	ATGGCAACCTACTGGATACG	External fragments of *ompR*
ompR-out-R	GAAGCCGCAAAGACACTG	
415-pDM4-F	TGGACAACAAGCCAGGGAT	Validation of recombinant pDM4 plasmid
415-pDM4-R	GCAAAGTGCGTCGGGTG	

Table S4 Primers for RT-qPCR to validate differentially expressed genes in *ΔompR*

Gene ID	Primers	Sequence (5'-3')	Notes
D3790_01075	01075-F	TATCTGGCTACATTGGAAA	109 bp
	01075-R	GCTCAAGGCTGGACTCAT	
D3790_02545	02545-F	TTGCTGCATTACTGGTGT	98 bp
	02545-R	TTCTACTGCTGTTGTGGG	
D3790_03530	03530-F	TTTTGCCTGTGCCTGAG	82 bp
	03530-R	GGTGCTGCTGTTTCTGG	
D3790_04500	04500-F	TGCTAGAAATGGGAATA	134 bp
	04500-R	TCTGCTGTATCGGTTTA	
D3790_04505	04505-F	TTATTATAGCCAGCAAAAC	98 bp
	04505-R	CTATGCGTAACCACAGC	
D3790_06365	06365-F	AATCACGGCAGTATCAG	150 bp
	06365-R	CAACGACACGAAACATC	
D3790_07005	07005-F	TGTTGGTGCCTGAGAAA	140 bp
	07005-R	GATAGTGGGCTTGATGG	
D3790_07420	07420-F	TATCCCTGTTCCTTGTTG	126 bp
	07420-R	TTGTCAGTTATTGCCTGT	
D3790_07425	07425-F	ACAATGCGTTCACTGCT	90 bp
	07425-R	ATGGGAAACACCCGATA	
D3790_08830	08830-F	TGCGTGAACTGGGATTG	98 bp
	08830-R	CTGTGCCGTCATTTGGA	
D3790_09100	09100-F	ACCAGAATCGGATTGGC	114 bp
	09100-R	GGTAGATAGGCAGGAACA	
D3790_10340	10340-F	ATCGTAGTGAGAATGGTGCTG	174 bp
	10340-R	CCACGCCTTCGGGTAA	
D3790_10545	10545-F	TGATGTGGCGAATGGGTTAC	92 bp
	10545-R	GCAGTGCCTGCCGAAAA	
D3790_10550	10550-F	GAGGGCATTGATGAACCG	93 bp
	10550-R	ACGCCAGTGACGACACGAT	
D3790_10580	10580-F	GGCTTTATTCCTGGTCC	126 bp
	10580-R	GAGATTGGCTTGCTCGA	
D3790_12470	12470-F	TGGTGCCTCATTAGTTC	105 bp
	12470-R	AAGCGTTCTGCTACTTT	
D3790_15845	15845-F	TGCCGTCCCTTTGCGTTAC	124 bp
	15845-R	TGCCGAGTCCAATCAGTTCA	
D3790_15850	15850-F	TTGTTCCAGCCCAAAGC	95 bp
	15850-R	TTTCGCCTGAGACACCC	
D3790_18675	18675-F	CCATTATCACGGGACTT	145 bp
	18675-R	GGGAGAACCTGCTTTGC	
D3790_18925	18925-F	GATACACTGTTCCGTATTGC	129 bp
	18925-R	AAACTCCTGCCCTTCCT	
D3790_02925	recA-F	CAGCGTGAAGGCAGAA	114 bp
	recA-R	GGTATCAGGCTGCGAAC	
